# Willingness to pay for community-based health insurance and associated factors among rural households of Bugna District, Northeast Ethiopia

**DOI:** 10.1186/s13104-019-4091-9

**Published:** 2019-01-24

**Authors:** Amare Minyihun, Measho Gebreslassie Gebregziabher, Yalemzewd Assefa Gelaw

**Affiliations:** 1 Department of Health Systems and Policy, Institute of Public Health, College of Medicine and Health Sciences, University of Gondar, P.o. Box: 196, Gondar, Ethiopia; 2 Department of Health Systems, School of Public Health, College of Health Sciences, Mekelle University, Mekelle, Ethiopia; 3 Department of Epidemiology and Biostatistics, Institute of Public Health, College of Medicine and Health Sciences, University of Gondar, Gondar, Ethiopia

**Keywords:** Community based health insurance, Willingness to pay, Tobit model, Bugna district, Ethiopia

## Abstract

**Objective:**

Community based health insurance schemes are becoming recognized as powerful method to achieve universal health coverage and reducing the financial catastrophic shock of the community. Therefore, this study aimed to assess willingness to pay for community-based health insurance and associated factors among rural households of Bugna District, Ethiopia.

**Results:**

A total of 532 study participants were included in the study. The finding indicated that 77.8% of the households were willing to pay for the community-based health insurance. The average amount of money the households were willing to pay per household per annum was 233 ETB ($11.12 USD). The result of the study also revealed that attending formal education[ß = 3.20; 95% CI = 1.87, 4.53], history of illness [ß = 2.52; 95% CI = 1.29, 3.75], household size [ß = 0.408; 95% CI = 0.092, 0.724], awareness about the scheme [ß = 2.96; 95% CI = 1.61, 4.30], and wealth status [ß = 5.55; 95% CI = 4.19, 6.90] were factors significantly associated with willingness to pay. Therefore, enhancing awareness of the community about the scheme, considering the amount of premium as per household family size and wealth status might increase household’s willingness to pay for community-based health insurance.

**Electronic supplementary material:**

The online version of this article (10.1186/s13104-019-4091-9) contains supplementary material, which is available to authorized users.

## Introduction

Globally, 150 million people per year suffer from financial catastrophic shock, and 100 million are pushed into poverty because of direct payments for health services. In the majority of African countries, more than 40% of their total health expenditure was constituted by out-of-pocket payment (OPP) and this resulted in scarcity of funds for health [1]. As a result of this, developing health financing system was a common agenda for all countries [2–4].

In Ethiopia OPP accounts 37% of the total spending in health care. This leads to financial catastrophic shock [5]. To overcome these challenges an efficient and equitable health care financing strategy was formulated [6]. The immediate goals of universal health coverage (UHC) are access, utilization and financial protection, while health status is a longer-term indirect result (although probably the ultimate goal), and one of the key strategies is reducing direct payments [2].

The government of Ethiopia has introduced Community Based Health Insurance (CBHI) as strategy for reducing financial catastrophic shock in our country, and it has been piloted in selected districts of the country. The utilization of scheme by the community members were affected by different factors such as socio-demographic, economic and health related factors [7].

A pilot study in Ethiopia has indicated that the scheme provides a benefit package of all available services in the health centres and hospitals excluding tooth implantation and eyeglasses with the provider payment mechanism of fee-for-service. Accordingly, the membership for the scheme could be all core family members [8, 9].

According to the Health Sector Financing Reform (HSFR) project report, the overall enrollment in the pilot schemes was 48%. It varies from district to district and ranges from 25% in Deder to 100% in Yirgalem, and households registered as indigents is 7%, and ranges from 1% in Deder to 15% in Tehuledere and Yirgalem [10].

Understanding the community’s willingness to pay (WTP) for the CBHI and determining the amount of money is useful for policy makers to implement the program and to expand the program nationally. Therefore, the objective of this study was to assess the WTP for CBHI and associated factors among rural households in Bugna District, Ethiopia.

## Main text

### Methods

Community based cross-sectional study was conducted from February to March, 2016 among households in Bugna district. Bugna district is found in Amhara National Regional State which is 354 km far from Bahir Dar (capital city of the region). The district has thirteen rural and one urban kebele. According to the district health office report, it has a population of 91,750 in 2016 [11]. The district has 4 health centers and 13 health posts that provide health service for the dwellers.

All households in the rural kebele of district were the source population whereas all households in the selected rural kebele of the district were the study population. Those permanent residents of the community with household head aged 18 years and above were included in the study. Households with heads or spouses that have been employed in the formal sectors were excluded from the study.

The sample size of the study was calculated using single population proportion formula with the assumption of; proportion of WTP for CBHI in Fogera district (p = 80%) [12]. The total sample size was 541 after considering 95% confidence level, 5% margin of error, design effect of 2 and 10% non-response rate.

Multi-stage systematic sampling technique was employed to select study participants. First, five out of 13 rural kebeles were selected using lottery method. Then, the sample was proportionally allocated among the selected kebeles based on the number of the households. Finally, systematic random sampling technique was used to select participants.

The dependent variable was WTP for CBHI, whereas socio- demographic characteristics (age, sex, marital status, educational status, family size, religion and ethnicity), economic factors(wealth index and occupation); environmental factors (distance from health institution in walking time);health and health-related factors (health condition of illness, chronic illness and disability, medical treatment for the recent episode, health care cost of the recent treatment, perceived quality of the health care service in the area); knowledge related factors(awareness about CBHI and social trust were independent variables of the study. The CBHI scheme (insurer) covers all costs of the members related to transportation, drugs, laboratory tests, hospitalization, and other necessary imaging examinations, but expenses of some selected chronic non-infectious diseases such as diabetes mellitus, heart failure, cancer, and treatment from abroad weren’t covered by the insurance. WTP for CBHI was measured using the bid contingent value method through asking a specific amount of money (180 ETB), and probing the question using a higher or lower bid value depending on the respondent’s response to the first question until the maximum amount of money participants were WTP. A pre-tested and standardized questionnaire was used to collect the data. Most of the tools were adapted from the previous studies of Benchi Maji [13] and Fogera [12]. The wealth item questions were adapted from the Ethiopian Health Insurance Agency CBHI evaluation report [8]. Pre- test was done on 10% of the subjects at Lasta District.

The collected data were cleaned, coded, entered into EPI-INFO version 7 software and exported and analysed using STATA software package version 14. Variables with p-value of less than 0.2 during bivariate analysis were considered for the multiple regression analysis [14–16]. Regression coefficient with 95% CI, t-value and p-value were used to measure the strength association.

Tobit model was used to identify factors associated with WTP and the maximum amount of money that individuals were WTP. This model has an advantage over other discrete choice models (linear, logistic, and probit) in that, it reveals both the probability of WTP and the maximum amount of money the respondents are WTP.

$${\text{y}} = \left\{ {\begin{array}{*{20}l} {1\quad if\;MWTP =\upbeta_{0} + {\beta^{\prime}}Xi + {\text{e}} > 0} \\ {0\quad if\;MWTP \le 0} \\ \end{array} } \right.$$

where y = outcome; MWTP = Maximum willingness to pay; Xi = explanatory variables; β_0_ = Slope, β′ = Coefficient; e = error term; 0 = No and 1 = yes.

The model also estimates marginal effect of an explanatory variable on the expected value of the dependent variable. A p-value = 0.05 was used to determine statistical significance.

### Results

#### Socio-demographic characteristics of households

A total of 532 participants participated in the study with response rate of 97.4%. The median age (IQR) of the respondents was 35 (14) years, ranging from 18 to 75 years. The majority of respondents were males (66%), farmer (70.3%), illiterate (55.3%). Moreover, 71.6% of the participants were married and the mean family size of the participants were 4.8 (± 1.8) (Table 1).

**Table 1:** Demographic and socio-economic characteristics of study participants in Bugna District, North East Ethiopia, 2016 (n = 532)

Variables	Description	Number	Percent (95% CI)
Sex of the respondent	Male	351	65.9 (61.8, 69.8)
	Female	181	34.1 (30.1, 38.1)
Age of the respondents	≤ 29 years	153	28.8 (25.1, 32.7)
	30–39 years	184	34.6 (30.6, 38.7)
	40–49 years	133	25.0 (21.4, 28.8)
	50–59 years	40	7.5 (5.5, 10.0)
	60 years and above	22	4.1 (2.7, 6.2)
Marital status of respondents	Single	38	7.1 (5.2, 9.6)
	Married	433	81.4 (77.8, 84.4)
	Windowed	8	1.5 (0.7, 2.9)
	Divorced	53	10.0 (7.6, 12.8)
Occupation of the respondents	Farmer	374	70.3 (66.2, 74.0)
	Housewife	82	15.4 (12.5, 18.7)
	Merchant	46	8.6 (6.5, 11.3)
	Daily labourer	8	1.6 (0.8, 3.0)
	Students	22	4.1 (2.7, 6.2)
Educational status	Unable to read and write	294	55.3 (50.9, 59.4)
	Read and write	122	29.9 (19.5, 26.7)
	Primary education	93	17.5 (14.4, 20.9)
	Secondary education	23	4.3 (2.8, 6.4)
Family size	≤ 5	308	57.9 (53.5, 61.9)
	> 5	224	42.1 (38.0, 46.4)
Household having children < 5 years old	No child	136	25.6 (22.0, 29.4)
	One child	250	47.0 (42.7, 51.2)
	2 and above	146	27.4 (23.8, 31.4)
Household having a person above 65 years old age	No older age	464	87.2 (84.0, 89.8)
	One and above	68	12.8 (10.1, 15.9)
Wealth quintile of the household	Poor (1st quintile)	172	32.3 (28.4, 36.4)
	Medium (2nd quintile)	182	34.2 (30.2, 38.3)
	Rich (3rd quintile)	178	33.5 (29.5, 37.5)

#### Health related characteristics of study participants

One hundred ninety-six (36.5%) respondents evaluated their family’s health status as medium. Eighty-seven (16.4%) of the member of the households had at least one member with chronic illness while 41.0% had at least one member who had encountered acute illnesses 3 months prior to data collection. The median expenditure of the 145 households which got treatments was 60 ETB with range of 10 to 1500 ETB. Sixty-eight (46.9%) of these households reported that it was difficult to cover payments for treatments. As a result, 43.4% of them have borrowed money from someone to cover their medical costs (Table 2).

**Table 2:** Health related characteristics of study participants in Bugna district, 2016

Variable	Description	Number	Percent (95% CI)
Self-reported health status of HHs (n = 532)	Very poor	11	2.1 (1.1, 3.7)
	Poor	72	13.5 (10.8, 16.7)
	Medium	196	36.5 (32.8, 41.0)
	Good	189	35.5 (31.5, 39.7)
	Very good	64	12.4 (9.5, 15.0)
Chronic illness (n = 532)	No	445	83.6 (80.2, 86.5)
	Yes	87	16.4 (13.4, 19.7)
Any illness during the past 3 months (n = 532)	No	314	59.0 (54.7, 63.1)
	Yes	218	41.0 (36.8, 45.2)
Get treatment (n = 218)	No	73	33.5 (29.5, 37.5)
	Yes	145	66.5 (58.6, 74.7)
Coverage of the health care cost (n = 145)	Self	135	93.1 (87.5, 96.2)
	Free/community or Gov.	10	6.9 (3.3, 11.9)
Satisfaction with health care service and costs (n = 145)	Very dissatisfied	4	2.7 (1.0, 7.1)
	Dissatisfied	11	7.6 (4.2, 13.2)
	Neutral	13	9.0 (5.2, 14.9)
	Satisfied	88	60.7 (52.4, 68.3)
	Very satisfied	29	20.0 (14.2, 27.3)
Perceived quality of the health care service in the district (n = 145)	Very low	3	2.0 (0.6, 6.2)
	Low	12	8.3 (4.7, 14.0)
	Medium	12	8.3 (4.7, 14.0)
	High	89	61.4 (53.1, 69.0)
	Very high	29	20.0 (14.2, 27.3)
Concern of the HH for covering health care costs (n = 145)	Very difficult	28	19.3 (13.6, 26.6)
	Difficult	68	46.9 (38.8, 55.1)
	Not difficult	49	33.8 (26.4, 41.9)
Borrow money for medical costs (n = 145)	No	82	56.6 (51.9, 60.4
	Yes	63	43.4 (33.8, 47.6)
Home distance from health centre or hospital in minutes	< 30 min	132	24.8 (21.3, 28.6)
	30-min to 1 h	185	34.8 (30.8, 38.9)
	1 h ≤ 2 h	121	22.7 (17.1, 29.9)
	> 2 h	94	17.7 (14.6, 21.1)

The mean amount of money household heads willing to pay was 233.3 ETB (95% CI = 225.6, 241.4) per house hold annually or 11.1 USD (Additional files 1 and 2).

#### Factors associated with willingness to pay for CBHI

For each an additional household member, the WTP value of households was increased by 0.4 USD other conditions being held constant (ß = 0.408, 95% CI (0.092, 0.724).

The study also revealed that respondents who attended formal education were WTP 3.2 USD more than those who didn’t attend formal education, holding other variables constant (ß = 3.20, 95% CI (1.87, 4.53).

Households who had awareness about the scheme were WTP 2.9 USD more than those who had no awareness about the scheme, holding other variables constant (ß = 2.96, 95% CI (1.61, 4.30).

Households who had history of illness in household member were WTP 2.5 USD more than the counter factual, holding other variables constant (ß = 2.52, 95% CI (1.61, 4.30). Similarly, households with higher economic status were WTP 5.5 USD more than those who are in lower socioeconomic status, holding other variables constant (ß = 5.55, 95% CI (4.19, 6.90) (Table 3; Additional file 3).

**Table 3:** Maximum likely hood of Tobit econometric analysis of factors associated with WTP for CBHI in Bugna district, 2016

Parameter for MWTP	Category	Unadjusted coefficient	adjusted coefficients	Standard error	t-value	p value	95% Conf. interval
Age	N	1.02	0.03	0.026	1.39	0.166	− 0.015, 0.007
Households family size	N	12.69	0.40	0.160	2.54	0.011*	0.092, 0.724
Educational status (ref. informal)	D						
Formal education		36.66	3.20	0.676	4.74	0.000*	1.87, 4.53
Awareness about the CBHIS (ref. poor awareness)	D						
Good awareness		75.25	2.96	0.685	4.32	0.000*	1.61, 4.30
Social trust among them (ref. poor trust)	D						
Good trust		7.42	1.20	0.756	1.60	0.111	− 0.27, 2.69
Chronic illness in household member (ref. no)	D						
Have chronic illness		11.62	− 0.05	0.804	-0.07	0.942	− 1.63, 1.52
History of any Illness in household member (ref. no)	D						
Have illness		19.30	2.52	0.626	4.03	0.000*	1.29, 3.75
Home distance from health centre or hospital in time (ref < 30 min)	D						
30-min to 1 h		− 23.15	− 2.71	1.850	− 1.46	0.144	− 6.34, 0.92
1 h ≤ 2 h		− 16.85	− 0.001	0.653	− 0.00	0.999	− 1.28, 1.28
> 2 h		11.32	0.99	0.827	1.21	0.229	− 0.62, 2.62
Perceived health status (ref poor)	D						
Medium		7.26	− 0.54	0.829	− 0.65	0.514	− 2.17, 1.08
Good		6.05	− 0.56	0.864	− 0.66	0.511	− 2.26, 1.13
Wealth quintile of the household (ref poor)	D		D				
Medium (2nd quintile)		27.22	2.10	0.668	3.14	0.002*	0.78, 3.41
Rich (3rd quintile)		85.00	5.55	0.691	8.03	0.000*	4.19, 6.90
Constant			− 0.93	1.59	− 0.59	0.559	− 4.07, 2.20
Sigma			5.90	0.21			5.48, 6.33

D, dummy variable (0, 1); N, numeric value

* Significant with p-value ≤ 0.05

### Discussion

This study aimed to assess WTP for CBHI and associated factors among households in Bugna district, Northeast Ethiopia. About 77.8% of participants were WTP for CBHI. The average amount of money WTP per household per annum was 233 ETB ($11.12 USD) for proposed CBHI. This implies the communities were WTP higher money than the proposed payment during the study period which was 140 up to 195 ETB. But the amount of money respondents WTP was consistent with the newly proposed premium which is 240. The mean amount of money WTP in the study is greater than study in Nigeria [17] and Ethiopia [9]. However, this was lower than study done in Cameroon [18], Nigeria [19], Namibia [20], Bangladesh [21] and Burkina Faso [22]. The discrepancy might be due to the difference in study period, area, design and participants.

The study revealed that households with larger family sizes were WTP a higher amount compared to households with smaller family size. This finding is supported by other studies conducted in Nigeria [17], Ethiopia [8], Bangladesh [21], china [23], and India [24]. The possible explanation might be as a result of the huge financial burden faced by households with a large size when they seek health care services.

Educational status of the respondents was found to be another factor to increase the amount of premiums WTP for CBHI scheme. This is consistent with a studies done in Nigeria [25], Cameron [18] and Burkina Faso [22]. Educated household heads have better knowledge about the advantage of making regular insurance payments to avoid the risk of catastrophic medical expenditures at the time of illness.

Respondent’s awareness about the CBHI had positive and significant association with WTP for the CBHI. This finding was consistent with studies conducted in Nigeria [26], Cameron [27], and Myanmar [28]. This might be due to knowing the catastrophic effects of health problem and the benefits of joining the insurance scheme earlier.

The wealth status of the families had positive and significant association with WTP. Which is similar with other studies conducted inEthiopia [12], Nigeria [19], Bangladesh [21], China [29], St. Vincent [30], and India [24]. The possible explanation might be having more wealth is associated with high asset losses if an unexpected event occurs that leads to be households more WTP for the insurance than the poorer.

The history of illness in the last 3 months in household members had significant effect to WTP for the CBHI. This finding was consistent with other studies in India [24], and Bangladesh [21]. This might be due to the risk-averse individuals are more likely to enrol in the insurance.

Political will and governmental commitment towards the scheme were the opportunities for CBHI implementation in the country. Accordingly, the presence of clear action plans, national scope of implementation and existence of regulatory frameworks were the strengths before introducing and start to implement the scheme. On the contrary, the decision for CBHI enrollment was made at the kebele level as opposed to the household level and a general subsidy from the federal government is also provided for all scheme members without giving especial emphasis for those members who cannot afford to pay. Furthermore, premium load was decided only by their family size without considering their level of income were the limitations of the scheme [8, 9].

Overall, this finding indicated that the proportion of households willing to pay for the scheme was low as compared to the goal of UHC. WTP for CBHI were influenced by educational status, history of illness, family size, awareness and wealth status of the households. Therefore, the government had better to consider the household family size and wealth status for setting the premium load. Moreover, strengthening awareness creation at the community about the scheme to enhance utilization of the scheme.

## Limitation

Even though this study lies on the large sample size with high response rate, the study has its own limitation on response biases which may overestimate or underestimate the results of WTP due to the use of self-reporting.

## Additional files

**Additional file 1: Figure S1.** Relationship ship of Maximum willingness to pay and the Premium level. In this study, As the premium level decreases the probability to pay for the community-based health increase. At low premium levels nearly, all study participants were willing to pay that premium or vice versa. The distribution of maximum willing ness to pay for the newly proposed community-based health insurance scheme in the rural households of Bugna district, 2016.

**Additional file 2: Table S1.** A bivariable with the proportion of WTP for CBHI and mean amount with 95% CI. In this study the mean amount of money willing to pay for CBHI for females’ respondents were 239.66 ETB, and age of respondent between 40 and 49 years were 240.29 ETB. The mean amount of WTP for CBHI for those respondents who have married, farmer, and unable to read and write were 239.91, 235.08 and 216.37 ETB, respectively. The mean amount of willing to pay of the proposed scheme for those respondents who have family member greater than five were 256.78; and those respondents who have been in the 3rd quantile of the wealth index were 302.42 ETB.

**Additional file 3: Table S2.** The marginal effect of factors associates with willingness to pay for CBHI. The result shows that when the family size of a household increases by one person, it will increase the probability of willingness of a household to pay for community-based health insurance by 1.12% [dy/dx = 0.0112, 95% CI (0.002, 0.020)]. The marginal effect of this variable reveals that respondents who had formal education 7.3% more probability of paying for community-based health insurance compared to respondents who did not have formal education [dy/dx = 0.0730, 95% CI (0.045, 0.100)]. The marginal effect of this variable reveals that respondents who had awareness about the scheme were 7.07% more probability of paying for community-based health insurance compared to respondents who did not have awareness about the scheme [dy/dx = 0.0707, 95% CI (0.043, 0.097)]. The marginal effect of this variable reveals that respondents who had history of illness in household member were 6.71% more probability of paying for community-based health insurance compared to respondents who did not have history of illness in the household member [dy/dx = 0.0671, 95% CI (0.034, 0.099)]. Marginal effect of factors associate with WTP for CBHI after Tobit econometrical analysis in Bugna district, 2016.

## Abbreviations

ANHB: Amhara Regional Health Bureau
CBHI: Community Based Health Insurance
CBHIS: Community-Based Health Insurance Scheme
DA: Developmental Agent
ETB: Ethiopian Birr
HEW: Health Extension Worker
NAD: Namibian Dollar
HSFR: Health Sector Financing Reform
OPP: out-of-pocket payment
UHC: Universal Health Coverage
$ USD: Untied States of American Dollar
VCHI: Voluntary Community Health Insurance
WTP: willingness to pay
