# Supplementary material for: Willingness to pay for community-based health insurance and associated factors among rural households of Bugna District, Northeast Ethiopia
Source: BMC Res Notes. 2019 Jan 24;12:55. doi: 10.1186/s13104-019-4091-9 (PMC6346545; doi:10.1186/s13104-019-4091-9)
Supplement: Supplementary file 1 — Additional file 1: Figure S1. Relationship ship of Maximum willingness to pay and the Premium level. In this study, As the premium level decreases the probability to pay for the community-based health increase. At low premium levels nearly, all study participants were willing to pay that premium or vice versa. The distribution of maximum willing ness to pay for the newly proposed community-based health insurance scheme in the rural households of Bugna district, 2016. [file 13104_2019_4091_MOESM1_ESM.pdf]

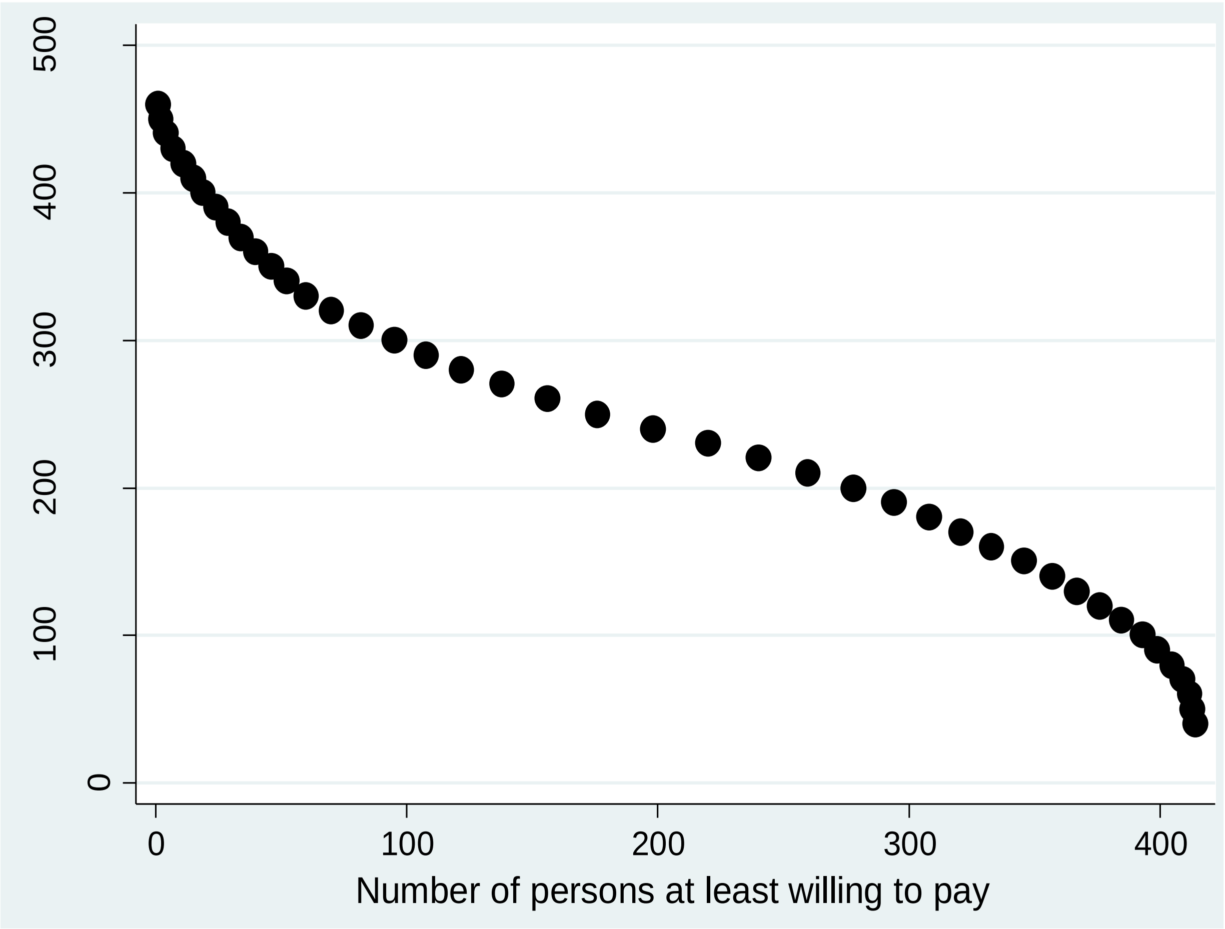

Fig.S1: the distribution of maximum willing ness to pay for the newly proposed community based health insurance scheme in the rural households of Bugna district, 2016
