# Supplementary material for: Willingness to pay for community-based health insurance and associated factors among rural households of Bugna District, Northeast Ethiopia
Source: BMC Res Notes. 2019 Jan 24;12:55. doi: 10.1186/s13104-019-4091-9 (PMC6346545; doi:10.1186/s13104-019-4091-9)
Supplement: Supplementary file 2 — Additional file 2: Table S1. A bivariable with the proportion of WTP for CBHI and mean amount with 95% CI. In this study the mean amount of money willing to pay for CBHI for females’ respondents were 239.66 ETB, and age of respondent between 40 and 49 years were 240.29 ETB. The mean amount of WTP for CBHI for those respondents who have married, farmer, and unable to read and write were 239.91, 235.08 and 216.37 ETB, respectively. The mean amount of willing to pay of the proposed scheme for those respondents who have family member greater than five were 256.78; and those respondents who have been in the 3rd quantile of the wealth index were 302.42 ETB. [file 13104_2019_4091_MOESM2_ESM.pdf]

Table S1: A bivariable of proportion of WTP for CBHI and mean amount with 95% CI

Variables	Description	Mean amount of MWTP in ETB (95% CI)	Proportion of WTP (mean amount of 233 ETB)	
			% of yes (95% CI)	% of No (95% CI)
Sex of the respondent	Male	221.65(207.27, 236.03)	82.25(76.04, 87.13)	17.75(12.86, 23.95)
	Female	239.66(229.49, 249.83)	73.86(63.61, 82.04)	26.14(17.95, 36.38)
Age of the respondents	≤ 29 years	221.46(206.40, 236.52)	74.02(63.00, 82.66)	25.98(17.33, 36.99)
	30-39 years	228.00(215.36, 240.63)	75.82(65.88, 83.58)	24.18(16.41, 34.11)
	40-49 years	240.29(221.74, 258.84)	83.07(71.80, 90.44)	16.93(9.5, 28.19)
	50-59 years	275.67(245.81, 305.53)	96.66(79.09, 99.5)	3.34(0.4, 20.90)
	60 years and above	228.23(185.12, 271.34)	81.81(47.25, 95.76)	18.19(4.2, 52.74)
Marital status of respondents	Single	199.64(176.24, 223.03)	66.67(39.58, 85.92)	33.33(14.07, 60.42)
	Married	239.91(230.65, 249.16)	81.81(76.26, 86.30)	18.19(13.69, 23.73)
	Windowed	233.33(161.61, 305.05)	60.00(16.74, 91.79)	40.00(8.2, 83.25)
	Divorced	203.95(177.06, 230.84)	69.56(47.86, 85.05)	30.44(14.94, 52.13)
Occupation of the respondents	Farmer	235.08(225.32, 244.84)	78.46(72.09, 83.70)	21.54(16.29, 27.90)
	Housewife	209.38(189.18, 229.58)	76.47(59.16, 87.93)	23.53(12.06, 40.83)
	Merchant	301.89(276.23, 327.55)	91.42(76.15, 97.27)	8.58(2.7, 23.84)
	Daily labourer	131.66(76.90, 186.42)	25.00(2.33, 57.43)	75.00(13.4, 97.33)
	Students	187.89(163.76, 212.02)	66.67(31.35, 89.74)	33.33(31.35, 89.74)
Educational status	Unable to read &write	216.37(206.39, 226.35)	72.59(64.40, 79.49)	27.41(20.50, 35.59)
	Read and write	241.83(223.02, 260.65)	84.48(72.59, 91.79)	15.52(8.2, 27.40)
	Primary education	267.44(247.09, 287.79)	88.40(78.34, 94.14)	11.60(5.85, 21.65)
	Secondary education	227.89(188.19, 267.59)	83.33(50.41, 96.09)	16.67(3.90, 49.58)
Family size	≤ 5	211.03(201.12, 220.95)	66.41(57.94, 73.95)	33.59(26.04, 42.05)
	Greater than 5	256.78(243.97, 269.58)	92.14(86.31, 95.61)	7.86(4.38, 13.68)
Wealth quintile of the household	Poor (1st quintile)	205.58(195.16, 216.00)	75.42(66.78, 82.40)	24.58(17.59, 33.21)
	Medium (2nd quintile)	241.60(281.87, 322.97)	79.16(69.79, 86.20)	20.84(13.79, 30.20)
	Rich (3rd quintile)	302.42(281.87, 322.97)	88.33(77.31, 94.38)	11.67(5.61, 22.68)
Chronic illness	No	231.56(222.06, 240.47)	78.66(72.78, 83.56)	21.34(16.43, 27.21)
	Yes	242.80(223.10, 262.49)	83.67(70.37, 91.70)	16.33(8.29, 29.62)
Awareness about the CBHIS	Poor awareness	218.83(210.44, 227.21)	76.09(69.73, 81.47	23.91(18.52, 30.26)
	Good awareness	294.00(273.47, 314.45)	89.85(80.06, 95.12)	10.15(4.87, 19.93)
Social trust among them	Poor trust	226.89(207.23, 246.55)	77.14(60.16, 88.29)	22.86(11.70, 39.83)
	Good trust	234.41(225.25, 243.56)	79.91(74.31, 84.54)	20.09(15.45, 25.68)
