# Supplementary material for: Willingness to pay for community-based health insurance and associated factors among rural households of Bugna District, Northeast Ethiopia
Source: BMC Res Notes. 2019 Jan 24;12:55. doi: 10.1186/s13104-019-4091-9 (PMC6346545; doi:10.1186/s13104-019-4091-9)
Supplement: Supplementary file 3 — Additional file 3: Table S2. The marginal effect of factors associates with willingness to pay for CBHI. The result shows that when the family size of a household increases by one person, it will increase the probability of willingness of a household to pay for community-based health insurance by 1.12% [dy/dx = 0.0112, 95% CI (0.002, 0.020)]. The marginal effect of this variable reveals that respondents who had formal education 7.3% more probability of paying for community-based health insurance compared to respondents who did not have formal education [dy/dx = 0.0730, 95% CI (0.045, 0.100)]. The marginal effect of this variable reveals that respondents who had awareness about the scheme were 7.07% more probability of paying for community-based health insurance compared to respondents who did not have awareness about the scheme [dy/dx = 0.0707, 95% CI (0.043, 0.097)]. The marginal effect of this variable reveals that respondents who had history of illness in household member were 6.71% more probability of paying for community-based health insurance compared to respondents who did not have history of illness in the household member [dy/dx = 0.0671, 95% CI (0.034, 0.099)]. Marginal effect of factors associate with WTP for CBHI after Tobit econometrical analysis in Bugna district, 2016. [file 13104_2019_4091_MOESM3_ESM.pdf]

Table S2: Marginal effect of factors associate with WTP for CBHI after Tobit econometrical analysis in Bugna district, 2016.

Parameter for MWTP	category	dy/dx	Std. Err.	Z	P>|z|	[95%CI.]
Age	N	0.0010	0.0007	1.38	0.169	-0.002, 0.002
Households family size	N	0.0112	0.0045	2.47	0.013*	0.002, 0.020
Educational status	D	0.0730	0.0139	5.26	0.000**	0.045, 0.100
awareness about the CBHIS	D	0.0707	0.0137	5.14	0.000**	0.043, 0.097
Social trust among them	D	0.0374	0.0252	1.48	0.138	-0.012, 0.087
Chronic illness in household member	D	-0.0023	0.0217	0.11	0.915	-0.040, 0.044
History of any Illness in household member	D	0.0671	0.0165	4.07	0.000**	0.034, 0.099
Home distance from health centre or hospital in time	D	0.0074	0.0072	1.03	0.303	-0.006, 0.021
Perceived health status	D	-0.0068	0.0116	-0.59	0.557	-0.029, 0.015
Wealth quintile of the household	D	0.0777	0.0113	6.87	0.000	0.055, 0.099

Note: - **significant with p-value ≤ 0.01; *significant with p-value ≤ 0.05; D= Dummy variable (0, 1); N=Numeric value;
